# Impact of type 2 diabetes mellitus on the long-term mortality in patients who were treated by coronary artery bypass surgery: A systematic review and meta-analysis

**DOI:** 10.1097/MD.0000000000007022

**Published:** 2017-06-02

**Authors:** Pravesh K. Bundhun, Akash Bhurtu, Jun Yuan

**Affiliations:** a Institute of Cardiovascular Diseases, The First Affiliated Hospital of Guangxi Medical University; b Guangxi Medical University; c Department of Cardiology, The People's Hospital of Guangxi Zhuang Autonomous Region, Nanning, Guangxi, PR China.

**Keywords:** cardiovascular diseases, coronary artery bypass surgery, mortality, type 2 diabetes mellitus

## Abstract

**Background:**

Recent scientific reports have mainly focused on the comparison between coronary artery bypass surgery (CABG) and percutaneous coronary intervention. However, the impact of type 2 diabetes mellitus (T2DM) on mortality in patients who were treated by CABG was often ignored. Therefore, we aimed to compare the long-term mortality following CABG in patients with and without T2DM.

**Methods:**

Studies comparing the long-term adverse outcomes following CABG in patients with and without T2DM were searched from electronic databases. Total number of deaths (primary outcome) and events of myocardial infarction (MI), major adverse cerebrovascular and cardiovascular events (MACCEs), stroke, and repeated revascularization (secondary outcomes) were carefully extracted. An analysis was carried out whereby odds ratios (ORs) and 95% confidence intervals (CIs) were calculated using the RevMan 5.3 software.

**Results:**

Eleven studies with a total number of 12,965 patients were included. Current results showed that mortality was significantly higher in patients with T2DM with OR: 1.54, 95% CI: 1.37 to 1.72, *P* < .00001; OR: 1.53, 95% CI: 1.36 to 1.72, *P* < .00001; and OR: 1.53, 95% CI: 1.26 to 1.87, *P* < .0001 at 1 to 15, 5 to 15, and 7 to 15 years, respectively. However, MI, repeated revascularization, MACCEs, and stroke were not significantly different with OR: 1.15, 95% CI: 0.81 to 1.64, *P* = .44; OR: 1.09, 95% CI: 0.88 to 1.36, *P* = .43; OR: 1.11, 95% CI: 0.83 to 1.48, *P* = .48; and OR: 1.69, 95% CI: 0.93 to 3.07, *P* = .08, respectively.

**Conclusion:**

Following CABG, a significantly higher rate of mortality was continually observed in patients with T2DM compared to patients without T2DM showing that the former apparently has a high impact on the long-term mortality. However, even if T2DM is an independent risk factor for mortality, it should not be ignored that CABG remains the best revascularization strategy in these patients.

## 1 Introduction

Type 2 diabetes mellitus (T2DM) is a major risk factor for cardiovascular diseases (CVDs).^[1]^ Several studies showed CVDs to have been responsible for more than 80% of death in patients with T2DM, and unfortunately, the actual number of patients with T2DM is estimated to double by the year 2030, indicating a rise in mortality during the coming years.^[2]^

Recent scientific reports comparing percutaneous coronary intervention (PCI) with coronary artery bypass surgery (CABG) in nondiabetic patients with multivessel coronary artery diseases who were good candidates for either procedure showed that PCI and CABG provided almost similar results following revascularization.^[3,4]^ However, in patients with T2DM, CABG was associated with better long-term clinical outcomes.^[5]^ It was suggested that CABG should be considered the revascularization procedure of choice in patients with T2DM who were complicated with multivessel coronary artery diseases.^[6]^

Recent scientific reports have mainly focused on the comparison between CABG and PCI. However, the impact of T2DM on mortality in patients who were treated by CABG was often ignored. Therefore, we aimed to compare the long-term mortality following CABG in patients with and without T2DM.

## 2 Methods

### 2.1 Data sources and search strategy

Electronic databases (Medline, EMBASE, and the Cochrane Library) were searched for randomized controlled trials (RCTs) and observational studies (English publications) comparing the long-term mortality following CABG in patients with and without T2DM. The words “coronary artery bypass surgery and diabetes mellitus” were the searched terms that were used. In addition, the abbreviations “CABG and DM” were also considered in this search strategy.

Apart from the abovementioned terms, the words “coronary artery bypass surgery” were also substituted by the words “surgical revascularization”. Reference lists of suitable articles were also reviewed for relevant publications.

### 2.2 Inclusion and exclusion criteria

Studies were included if:

(1) They were RCTs or observational studies comparing the adverse clinical outcomes following CABG in patients with and without T2DM.
(2) They reported mortality among their clinical endpoints.
(3) They had a follow-up period of 1 or more years.

Studies were excluded if as follows:

(1) They were meta-analyses, case studies, or letters to editors.
(2) They had a shorter follow-up period (<1 year).
(3) They did not report mortality among their clinical endpoints.
(4) They involved patients who were revascularized by CABG without the inclusion of a control group.
(5) They were duplicate studies.

### 2.3 Outcomes, definitions, and follow-ups

The primary endpoint was mortality and the secondary endpoints were the other adverse cardiovascular outcomes which have been listed below.

These clinical endpoints included the following:

(1) Mortality: consisting of all-cause mortality and cardiac death.
(2) Myocardial infarction (MI).
(3) Stroke.
(4) Major adverse cerebrovascular and cardiovascular events (MACCEs). MACCEs were composed of death, MI, stroke, and repeated revascularization.
(5) Repeated revascularization (target vessel revascularization and target lesion revascularization).

This analysis had a long-term follow-up period (1–15 years). The reported outcomes with their corresponding follow-up periods have been listed in Table 1.

**Table 1:**
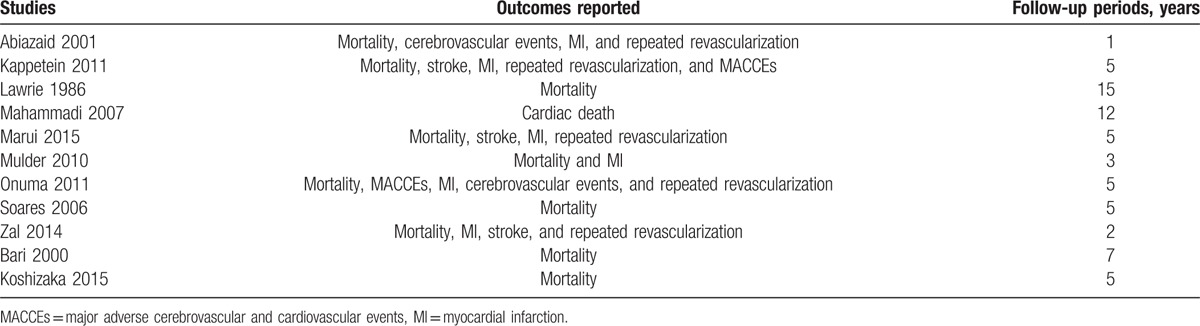
Reported outcomes and follow-up periods.

### 2.4 Data extraction and review

Two authors (PKB and AB) reviewed the relevant articles and carefully assessed the eligibility criteria of each included studies. Information and data concerning the types of study reported the total number of patients with and without T2DM, respectively, the primary and secondary endpoints, as well as data relevant to the baseline features of the patients were carefully extracted. Any disagreement concerning data extraction was solved by the third author (JY). Bias risk was assessed with reference to the Cochrane Collaboration.^[7]^

### 2.5 Statistical analysis

The Preferred Reporting Items for Systematic Reviews and Meta-Analyses guideline was reported.^[8]^ Possible heterogeneity among the subgroups analyzing the primary and secondary outcomes was assessed using the Cochrane *Q*-statistic and the *I*^2^-statistic tests, respectively. According to the *Q*-statistic test, a result with *P* value ≤.05 was considered statistically significant.

The *I*^2^ value also had a major role in this analysis. Heterogeneity increased with a rising *I*^2^ value. That is, the larger the *I*^2^ value, the higher the heterogeneity. If *I*^2^ corresponded to a value less than 50%, a fixed effects model was used. However, if *I*^2^ corresponded to a value greater than 50%, a random effects model was used.

The main analysis was carried out whereby odds ratios (ORs) and 95% confidence intervals (CI) were calculated using the RevMan 5.3 software.

Sensitivity analyses were also carried out by excluding each study one-by-one, and a new analysis was repeated each time.

Since this analysis did not include many trials/observational studies, publication bias was visually estimated by visually assessing funnel plots that were obtained directly from the RevMan software.

Ethical approval was not necessary for this type of study.

All the 3 authors had full access to the data, and they approved the manuscript as written.

## 3 Results

### 3.1 Search outcomes

**Figure 1:**
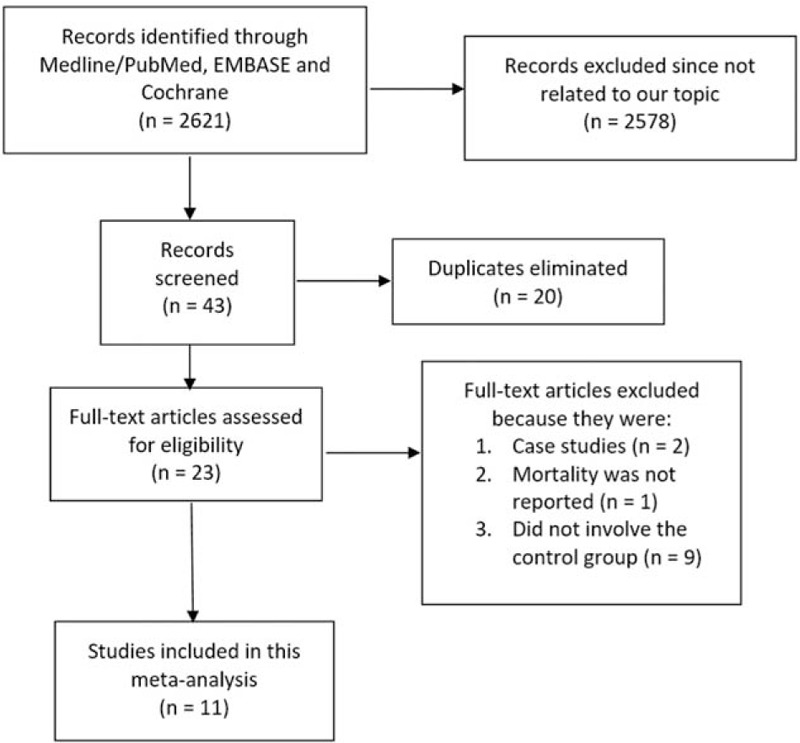
Flow diagram representing the study selection.

A total of 2621 articles were obtained during the search process. After reviewing/assessing the titles and abstracts, 2578 articles were directly eliminated. Among the 43 remaining articles, 20 duplicated articles were further excluded. A total of 23 full-text articles were assessed for eligibility. A total of 12 publications were further eliminated because of the following reasons: case studies (2), they did not report mortality as their clinical endpoint (1), and they compared CABG with PCI in patients with T2DM without comparing the outcomes in patients without T2DM (9). Finally, only 11 studies (6 RCTs^[9–14]^ and 5 observational studies^[15–19]^) were included in this meta-analysis as shown in Fig. 1.

### 3.2 General features of the studies which were included

A total number of 12,965 patients were included in this analysis (4106 patients with T2DM and 8859 patients without T2DM). The general features of the studies which were included in this analysis have been summarized in Table 2.

**Table 2:**
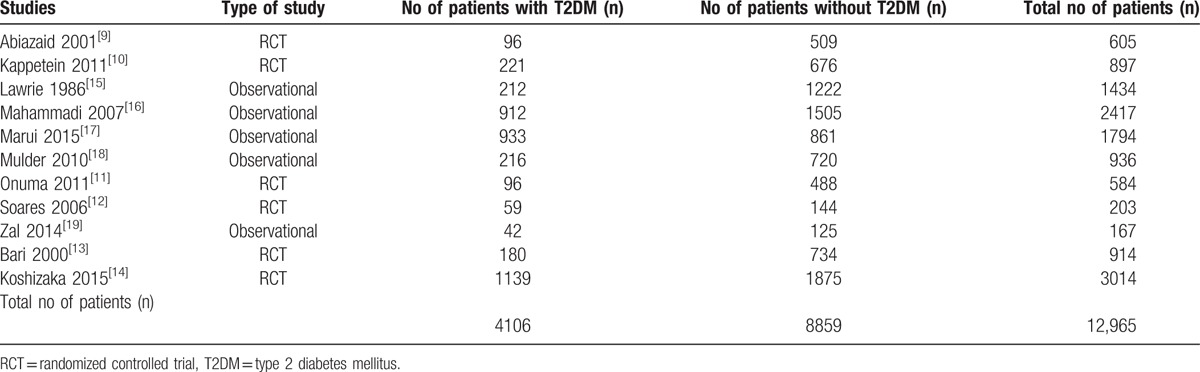
General features of the studies which were included.

Number of studies with: 1-year follow-up: 1; 2-year follow-up: 1; 3-year follow-up: 1; 5-year follow-up: 5; 7-year follow-up: 1; 12-year follow-up: 1; 15-year follow-up: 1.

### 3.3 Baseline features of the patients

The baseline features of the patients have been summarized in Table 3.

**Table 3:**
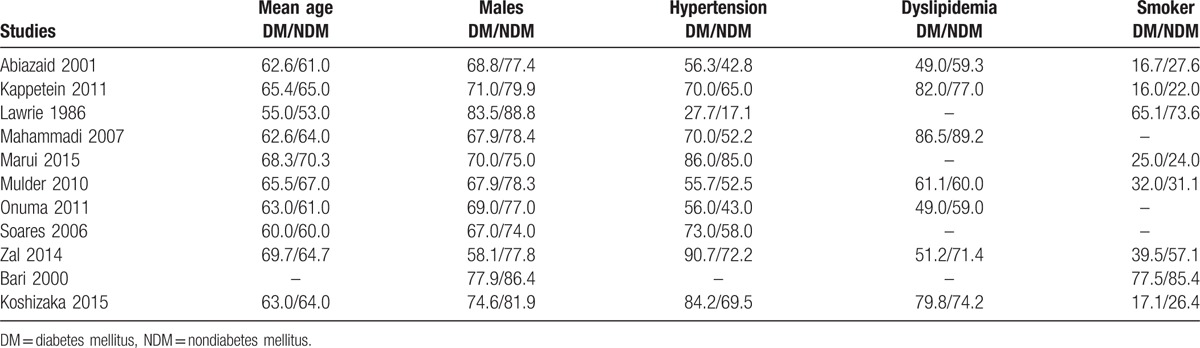
Baseline features.

The mean age was reported in years, whereas the other features were reported in terms of percentage (%). Mean age of the patients varied between 53.0 and 70.3 years. Majority of the patients in study Marui2015 within both groups had hypertension, whereas study Mahammadi2007 consisted of most patients with dyslipidemia. Overall, except for hypertension that was more prominent in patients with T2DM, there was no other major significant difference in baseline features between patients with and without T2DM who were enrolled in this analysis.

### 3.4 Mortality analyzed

**Figure 2:**
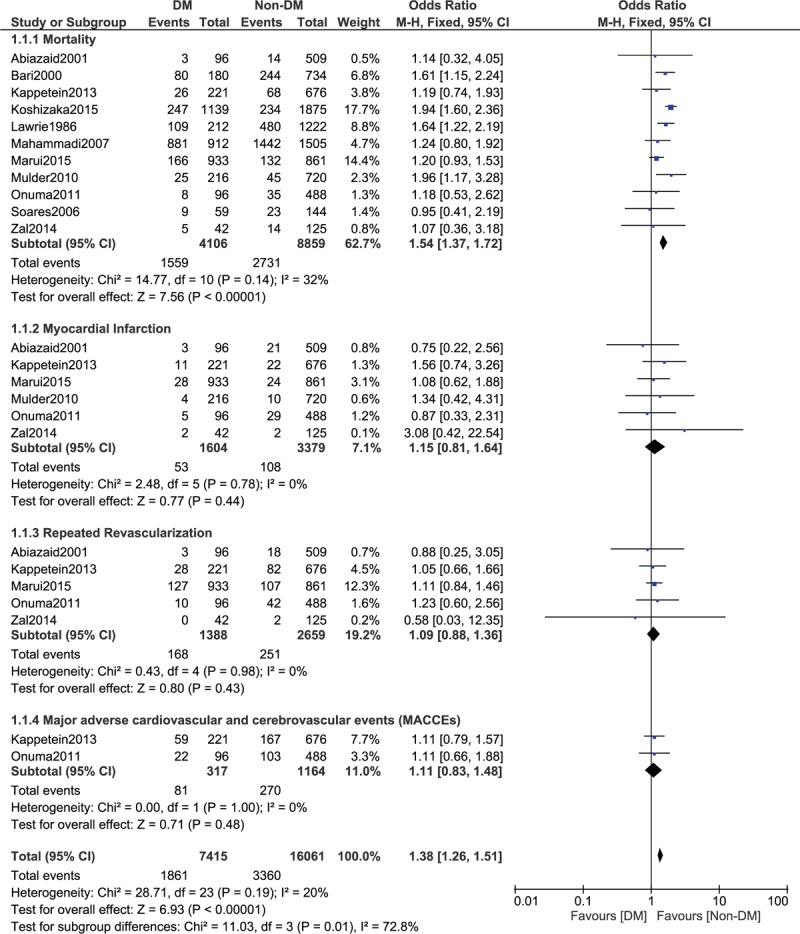
Mortality and adverse cardiovascular outcomes reported between type 2 diabetes mellitus (T2DM) and non-T2DM following revascularization by coronary artery bypass surgery (1–15 years).

Results of this study showed that among the 12,965 patients who were analyzed for all-cause death (4106 patients with T2DM and 8859 patients without T2DM), mortality was significantly higher following CABG in patients with T2DM, with OR: 1.54, 95% CI: 1.37 to 1.72; *P* < .00001, *I*^2^ = 32% during the long-term follow-up period (1–15 years). Since a low level of heterogeneity (<40%) was reported in this subgroup, a fixed effects model was used during the statistical analysis. This result has been represented in Fig. 2.

**Figure 3:**
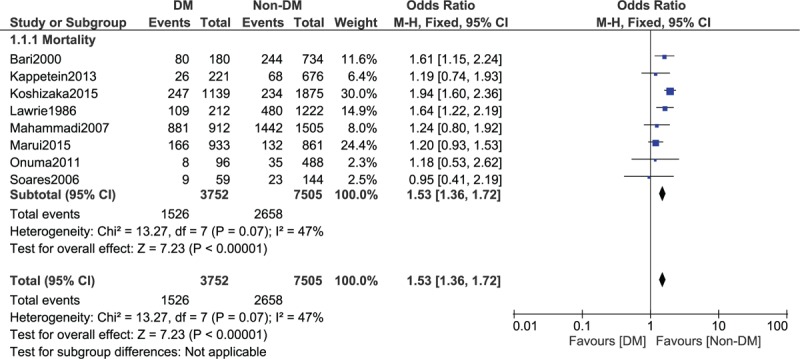
Long-term mortality observed between type 2 diabetes mellitus (T2DM) and non-T2DM following revascularization by coronary artery bypass surgery (5–15 years).

**Figure 4:**
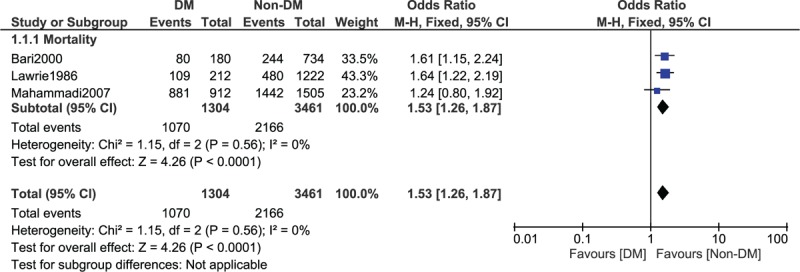
Long-term mortality observed between type 2 diabetes mellitus (T2DM) and non-T2DM following revascularization by coronary artery bypass surgery (7–15 years).

When a follow-up period of 5 to 15 years was considered, mortality was still significantly higher in patients with T2DM, with OR: 1.53, 95% CI: 1.36 to 1.72; *P* < .00001, *I*^2^ = 47% as shown in Fig. 3. A similar result was again obtained when a follow-up period of 7 to 15 years was considered, with OR: 1.53, 95% CI: 1.26 to 1.87; *P* < .0001, *I*^2^ = 0% as shown in Fig. 4.

**Figure 5:**
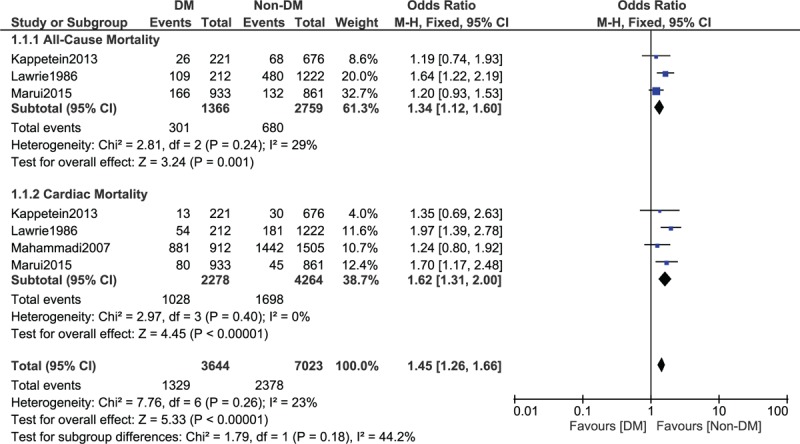
All-cause mortality and cardiac death observed between type 2 diabetes mellitus (T2DM) and non-T2DM following revascularization by coronary artery bypass surgery.

Several studies reported mortality as their outcome without clearly specifying whether it was all-cause mortality or cardiac death. Therefore, another subgroup analysis was carried out only with studies which specified the type of mortality which was reported. Results of this specific subgroup analysis showed all-cause mortality and cardiac death to significantly favor patients without T2DM with OR: 1.34, 95% CI: 1.12 to 1.60; *P* = .001 and OR: 1.62, 95% CI: 1.31 to 2.00; *P* < .00001, respectively, during this long-term follow-up (Fig. 5).

**Figure 6:**
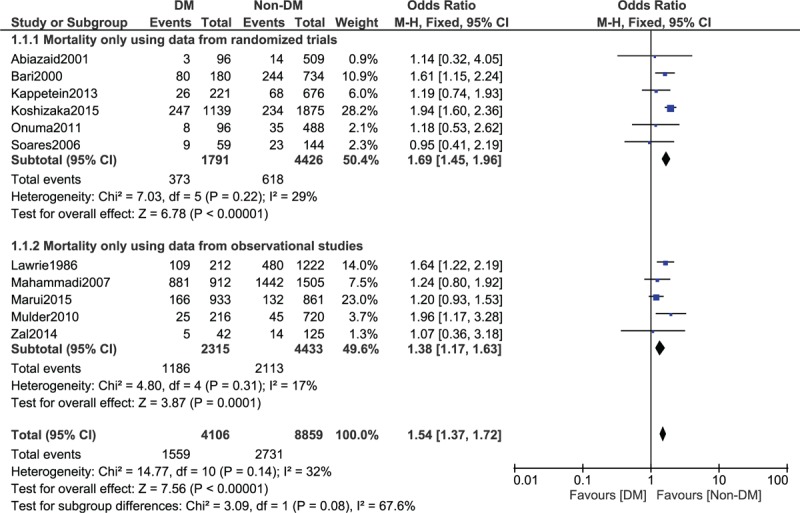
Randomized controlled trials and observational studies analyzed separately for long-term mortality.

This analysis was composed of data that were obtained from randomized trials and observational studies. However, separate analyses were further carried out using data that were obtained from randomized trials and observational studies, respectively. The results that were obtained showed mortality to still be significantly higher in patients with T2DM, OR: 1.69, 95% CI: 1.45 to 1.96; *P* < .00001 and OR: 1.38, 95% CI: 1.17 to 1.63; *P* = .0001, respectively (Fig. 6).

**Figure 7:**
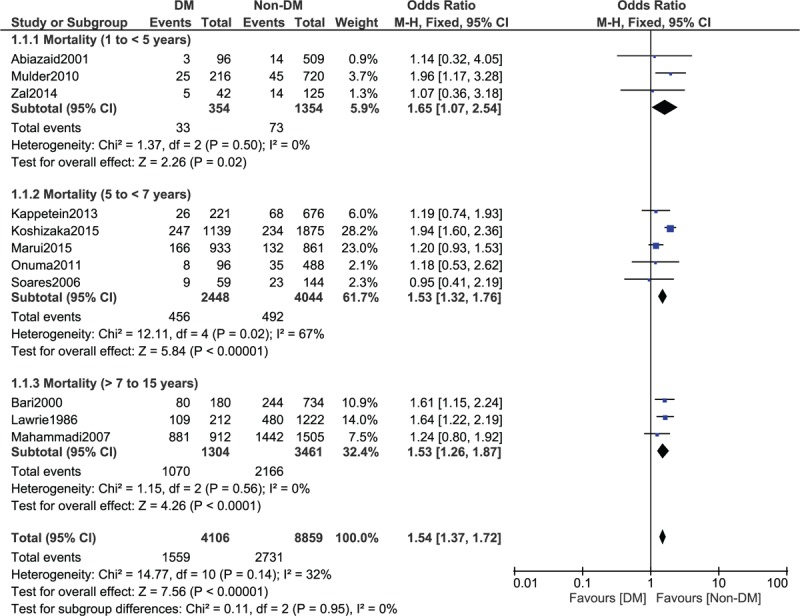
Mortality at different time period observed between type 2 diabetes mellitus (T2DM) and non-T2DM following revascularization by coronary artery bypass surgery.

Mortality was further subdivided into a short-term (1 year ≤ mortality < 5 years), middle-term (5 years ≤ mortality < 7 years), and long-term (7 years ≤ mortality ≤ 15 years) follow-ups. Significantly higher mortality rates were observed in patients with T2DM, OR: 1.65, 95% CI: 1.07 to 2.54, *P* = .02; OR: 1.53, 95% CI: 1.32 to 1.76, *P* < .00001; and OR: 1.53, 95% CI: 1.26 to 1.87, *P* < .0001 during a short-term, mid-term, and long-term follow-up periods, respectively (Fig. 7).

### 3.5 Other adverse cardiovascular outcomes that were analyzed

In this study, we also analyzed the other cardiovascular outcomes which were reported. Following CABG, MI, repeated revascularization, and MACCEs were not significantly different in patients with and without T2DM with OR: 1.15, 95% CI: 0.81 to 1.64, *P* = .44, *I*^2^ = 0%; OR: 1.09, 95% CI: 0.88 to 1.36, *P* = .43, *I*^2^ = 0%; and OR: 1.11, 95% CI: 0.83 to 1.48, *P* = .48, *I*^2^ = 0%, respectively, as shown in Fig. 2.

**Figure 8:**
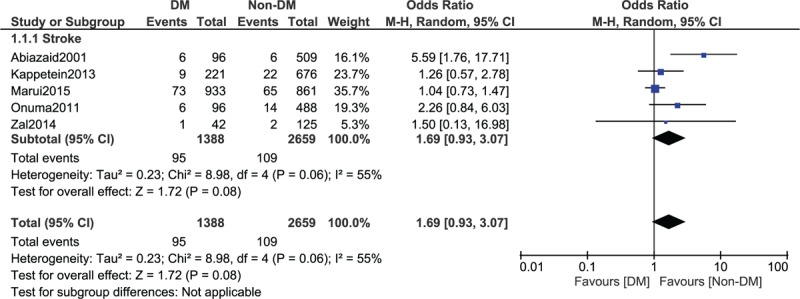
Stroke reported between type 2 diabetes mellitus (T2DM) and non-T2DM following revascularization by coronary artery bypass surgery (1–15 years).

However, even if stroke was higher in the diabetic group with OR: 1.69, 95% CI: 0.93 to 3.07; *P* = .08, *I*^2^ = 55%, the result was not statistically significant (Fig. 8).

The main result of this current analysis has been summarized in Table 4.

**Table 4:**
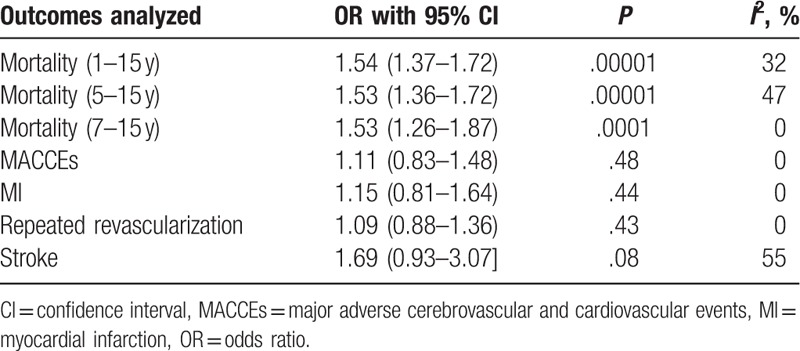
Results of this analysis.

**Figure 9:**
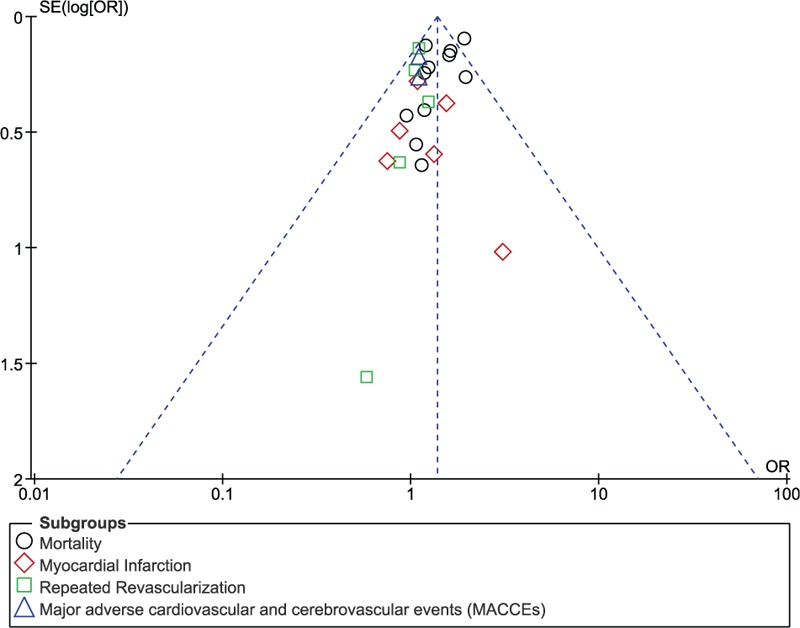
Funnel plot showing publication bias of 4 subgroups.

**Figure 10:**
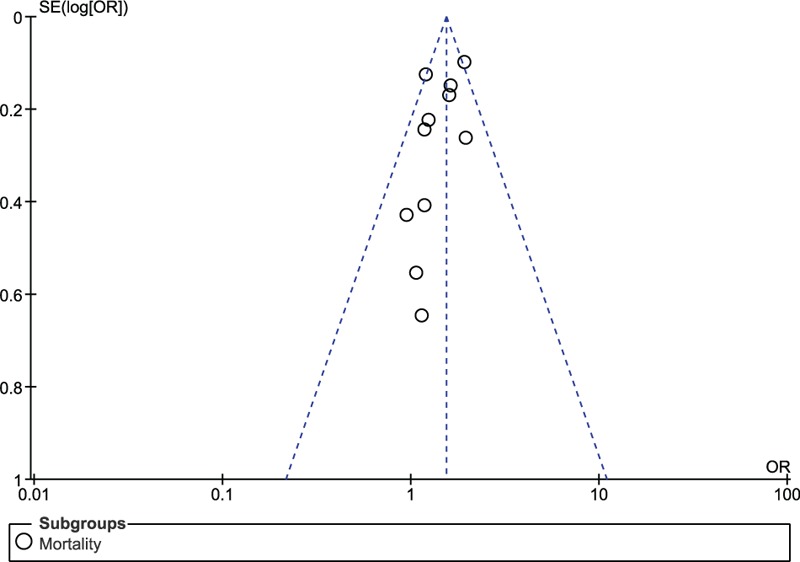
Funnel plot showing publication bias of 1 subgroup.

Sensitivity analyses yielded consistent results. In addition, there has been only little evidence of publication bias observed across all the studies which were involved in assessing the primary and secondary endpoints (Figs. 9 and 10).

## 4 Discussion

Even though T2DM is considered as a major risk factor contributing to the development of CVDs, the relationship between CVDs and T2DM is complicated.^[20,21]^

This study aimed to show the impact of T2DM on the long-term mortality following CABG. The current results showed mortality to have been consistently higher in patients with T2DM following CABG. Even when data that were obtained from randomized trials and observational studies were separately analyzed, T2DM was still associated with a significantly higher mortality rate. When the other cardiovascular outcomes were compared between patients with and without T2DM, no significant differences were observed among the subgroups analyzing MI, MACCEs, and repeated revascularization. However, following CABG, even if a higher rate of stroke was observed in patients with T2DM, the result was not statistically significant.

Even if all the data that were obtained have contributed significantly to the final results, the weight of each study was different during this analysis. When mortality was analyzed, study Bari2000 had a weight of 11.6%, study Kappetein2013 had a weight of 6.4%, study Koshizaka2015 had a weight of 30.0%, study Lawrie1986 had a weight of 14.9%, study Mahammadi2007 had a weight of 8.0%, and study Marui2015 had a weight of 24.4%. However, even excluding each study one-by-one, or even excluding the study apparently with the highest weight did not affect the results that were previously obtained. Therefore, it was clear that the main result that was obtained was not influenced by the result of 1 particular study.

Previously, when CABG was compared with PCI in patients with T2DM, PCI was associated with a significantly higher rate of repeated revascularization.^[22]^ However, a lower revascularization rate was reported following CABG, as reflected in this current analysis. Despite being higher in patients with T2DM, the result representing stroke was not statistically significant. To further support this point, Bundhun et al also recently compared PCI and CABG in patients with insulin-treated diabetes mellitus (ITDM). Their result showed CABG to be associated with a higher rate of stroke without statistically significance.^[6]^ However, when the same outcome was compared in patients with ITDM and noninsulin-treated T2DM (NITDM), a significantly higher rate of stroke was associated with insulin therapy.^[23]^ This current analysis only showed an insignificantly high rate of stroke observed among patients with T2DM. To note, this present study involved patients with T2DM as a whole, including patients with ITDM and NITDM combined together thus showing a slightly different result compared to the previously mentioned study. Munnee et al^[23]^ also showed a significantly higher rate of mortality and major adverse events associated with ITDM. However, results for MACCEs in this current analysis differed from theirs could be due to the fact that patients with T2DM were combined and analyzed without further being classified into ITDM and NITDM.

Koshizaka et al^[14]^ also showed T2DM to be associated with increased mortality compared to non-T2DM after 5 years following CABG. It should be noted that data from the PRoject of Ex-vivo Vein graft ENgineering via Transfection IV Trial (PREVENT IV) were used. In addition, a subgroup of the patients was monitored on the basis of whether they had insulin therapy before CABG or not. Several studies have also shown significantly higher mortality rate to be associated with T2DM patients on insulin treatment.

Reasons that have been suggested for this higher rate of adverse cardiovascular outcomes associated with insulin therapy were: more aggressive disease or several health complications presented in these patients, with a more advanced stage of T2DM.^[24]^ In addition, adverse effects of insulin could also be another reason contributing to a higher rate of mortality in patients with ITDM.^[24]^ Also, iatrogenic hyperinsulinemia promoting proinflammatory macrophage response and stimulating hormonal hyperactivation of signal transduction pathway,^[25,26]^ endogenous hyperinsulinemia which could increase hepatic synthesis of cholesterol^[27]^ could all be mechanisms suggested to contribute to this high rate of adverse events in this particular subgroup of T2DM patients.^[28,29]^ Unfortunately, this current analysis had limited data on patients with ITDM.

The retrospective study by Carson et al^[30]^ which aimed to show the impact of T2DM on the short-term mortality and morbidity in patients undergoing CABG indicated that T2DM was an important risk factor in patients undergoing CABG. Their study, which involved 434 hospitals in North America and included 41,663 patients with T2DM and 105,123 patients without T2DM, had a follow-up period of only 30 days. This current analysis showed T2DM to be independently associated with a long-term mortality following CABG in different subgroups of patients.

The study by Banning et al^[31]^ that compared the outcomes between CABG and paclitaxel eluting stents showed a comparable result among patients with and without T2DM who were revascularized by CABG in terms of composite endpoints including stroke, MI, and death altogether. However, the results of this present study showed death to significantly be lower in patients without T2DM, whereas MI was comparable between these 2 groups while stroke, despite being higher in the diabetic group, was not statistically significant.

Nevertheless, a few studies support the fact that patients with T2DM who were revascularized by CABG had substantially higher risk of major adverse events especially among ITDM.^[32]^ This present study reported a significantly higher rate of mortality in patients with T2DM following CABG, but however, results for MACCEs were not statistically significant and involved only 2 studies for comparison which was not sufficient to reach a conclusion in terms of this particular outcome. Results from the CABG Patch Trial Database showed that during a follow-up period of 4 years, T2DM was not a predictor of mortality after CABG.^[33]^ But the Trial involved only patients with left ventricular dysfunction with several comorbidities. However, in this current analysis, majority of patients with T2DM also suffer from hypertension which could have also influenced the mortality rate following CABG.

Even if T2DM has a high impact on mortality in patients undergoing revascularization with CABG during the long-term, in comparison to patients without T2DM, CABG remains the most effective revascularization procedure in patients with T2DM.^[10]^ Mortality might have also been attributable to the age of the patients and their clinical conditions. And when compared to PCI, neither revascularization procedure could completely eliminate attacks of the heart but were more effective compared to medical therapy.^[12]^ However, since a significantly higher repeated revascularization rate was observed with PCI, and due to the fact that CABG could restore blood flow to a larger extent, and blockade sites could be more accessible compared to PCI, CABG should be considered more effective in patients with T2DM. However, after revascularization with CABG, precautions such as implementing a heart healthy lifestyle, increasing regular physical exercises, smoking cessation, blood pressure and cholesterol control with diet and medications, and weight loss should all be considered in these T2DM patients with CVDs.^[34]^

## 5 Novelty

This study is new in the way that it is among the first meta-analyses showing the impact of T2DM on the long-term mortality rate in patients who were treated by CABG. Moreover, this study included a larger number of patients which were extracted from randomized trials and observational studies. Previous studies did not involve such a large number of patients. Also, this analysis compared long-term mortality during different sets of follow-up periods, and even separately compared mortality using data which were obtained from randomized trials and observational studies respectively. Cardiac death was also separately analyzed. A low level of heterogeneity among the different subgroups which were analyzed could also contribute to the novelty in this study.

## 6 Limitations

This study has limitations. First of all, due to the limited number of patients analyzed, this study might not generate very good results. Also, a moderate level of heterogeneity was observed when analyzing mortality during a follow-up period between 1 and 15 years, and 5 to 15 years, respectively. This could have been partly due to the involvement of data which were obtained from observational studies. In addition, this analysis was limited only to English publications which could lead to the introduction of selection and publication bias. Moreover, only 1 study had a follow-up period of 1, 2, 7, 12, and 15 years, respectively. The remaining studies had a follow-up period of 3 and 5 years, respectively. Therefore, even if a follow-up period ranging from 1 to 15 years was considered justified, it was limited to different follow-up periods. However, this point was further improved when an analysis considering a short-term (1 to < 5 years), mid-term (5 to < 7 years), and long-term (7 to 15 years) follow-up was carried out. Moreover, a high percentage of T2DM also suffered from hypertension showing that the latter might have partly contributed to this high mortality rate. Also, the drug history (cardiac medications used) prior and post CABG were not known. These medication uses could have had an influence on the results too. In addition, the large gap between study Lawrie1986 and the other studies might be a possible confounder because of significant recent advances in cardiovascular surgeries as compared to the year 1986. However, fortunately excluding study Lawrie1986 did not affect the results of this analysis. Finally, due to limited data reporting the number of patients on insulin therapy, we could not carry out another subgroup analysis based only on patients who were being treated by insulin.

## 7 Conclusion

Following CABG, a significantly higher rate of mortality was continually observed in patients with T2DM compared to patients without T2DM showing that the former apparently has a high impact on the long-term mortality. However, even if T2DM is an independent risk factor for mortality, it should not be ignored that CABG remains the best revascularization strategy in these patients.
